# Highly tunable hybrid metamaterials employing split-ring resonators strongly coupled to graphene surface plasmons

**DOI:** 10.1038/ncomms9969

**Published:** 2015-11-20

**Authors:** Peter Q. Liu, Isaac J. Luxmoore, Sergey A. Mikhailov, Nadja A. Savostianova, Federico Valmorra, Jérôme Faist, Geoffrey R. Nash

**Affiliations:** 1 Institute for Quantum Electronics, Department of Physics, ETH Zurich, Zurich CH-8093, Switzerland; 2 College of Engineering, Mathematics and Physical Sciences, University of Exeter, Exeter EX4 4QF, UK; 3 Institute of Physics, University of Augsburg, Augsburg 86159, Germany

## Abstract

Metamaterials and plasmonics are powerful tools for unconventional manipulation and harnessing of light. Metamaterials can be engineered to possess intriguing properties lacking in natural materials, such as negative refractive index. Plasmonics offers capabilities of confining light in subwavelength dimensions and enhancing light–matter interactions. Recently, the technological potential of graphene-based plasmonics has been recognized as the latter features large tunability, higher field-confinement and lower loss compared with metal-based plasmonics. Here, we introduce hybrid structures comprising graphene plasmonic resonators coupled to conventional split-ring resonators, thus demonstrating a type of highly tunable metamaterial, where the interaction between the two resonances reaches the strong-coupling regime. Such hybrid metamaterials are employed as high-speed THz modulators, exhibiting ∼60% transmission modulation and operating speed in excess of 40 MHz. This device concept also provides a platform for exploring cavity-enhanced light–matter interactions and optical processes in graphene plasmonic structures for applications including sensing, photo-detection and nonlinear frequency generation.

## Abstract (web summary)

Realizing tunable metamaterials across a broad spectral range is of great interest. Here, Liu *et al*. introduce hybrid structures comprising graphene plasmonic resonators strongly coupled to conventional split-ring resonators and reach 60% transmission modulation with an operating speed above 40 MHz.

## Graphical abstract

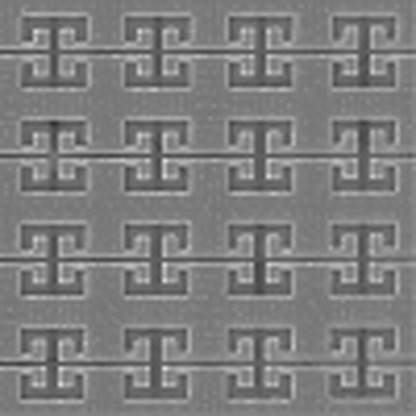

Since the turn of the century, research on metamaterials has progressed rapidly with substantial expansion of both the scope of novel functionalities and the operating frequency range enabled by different types of artificial structures^1^,^2^,^3^,^4^,^5^,^6^,^7^,^8^,^9^,^10^,^11^. Many of the demonstrated metamaterials are based on noble metals to take advantage of their negative permittivity below the plasma frequency. Real-time tunability of metamaterials is highly desired for many applications such as optical switches and modulators; however, it is a property lacking in metals. Different approaches have been developed to achieve tunable or reconfigurable metamaterials, among which several most effective realizations are based on changing the substrate properties^12^,^13^,^14^. Such approaches may find limitations where the properties of the substrate material cannot or should not be changed significantly, and metamaterial structures with intrinsic tunability^15^,^16^,^17^ are keenly sought after. Graphene, a more recently discovered and intensively studied material with various interesting properties such as its tunable carrier density and high room temperature carrier mobility, is a promising candidate for realizing tunable metamaterials across a broad spectral range^18^. Graphene's capability to support tightly confined surface plasmon (SP) in the THz to mid-infrared spectral range^19^,^20^ has been systematically investigated using scanning near-field optical microscopy^21^,^22^,^23^,^24^,^25^ and demonstrated in various patterned graphene structures^26^,^27^,^28^,^29^,^30^,^31^,^32^,^33^, including arrays of closely packed graphene ribbons (GR), that are essentially tunable metamaterials. However, the limited interaction between incident light and SP in monolayer graphene structures is not sufficient for many applications.

Here, we demonstrate as proof-of-concept a type of electrostatically tunable hybrid metamaterial employing graphene plasmonic resonators strongly coupled to conventional metal-based metamaterials. In addition to their strong electromagnetic response and high tunability, such hybrid metamaterials also provide an interesting platform for exploring cavity-enhanced optical processes and light–matter interactions in graphene plasmonic structures for applications including sensing^34^, photo-detection^35^,^36^ and nonlinear frequency generation^37^.

## Results

### Design principle of the hybrid metamaterials

**Figure 1:**
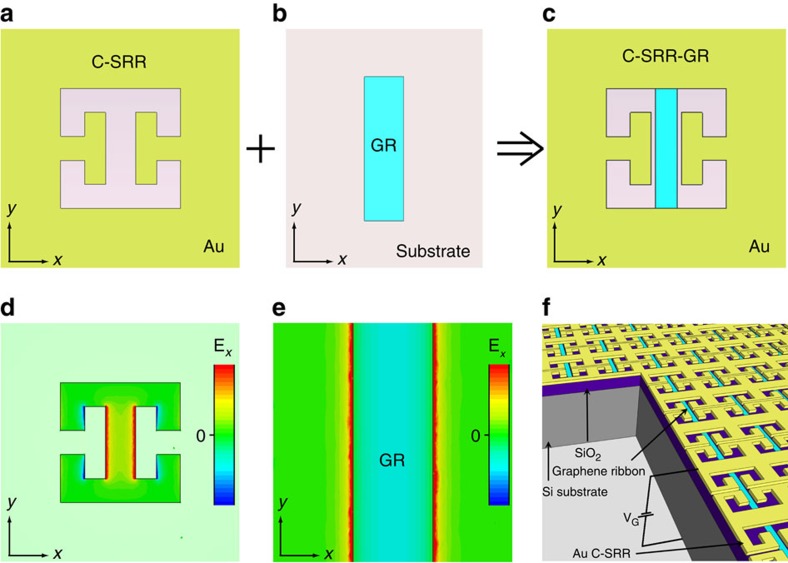
Design of the tunable C-SRR-GR hybrid metamaterials. (**a**) Schematic representation of a typical C-SRR. (**b**) Schematic representation of a GR on a substrate. (**c**) Schematic representation of a typical unit cell of the proposed C-SRR-GR hybrid metamaterials. (**d**) Simulated *x*-component of the E-field distribution associated with the LC-resonance of the C-SRR in **a**. (**e**) Simulated *x*-component of the E-field distribution associated with the localized SP resonance of the GR in **b**. (**f**) Three-dimensional schematic representation of the proposed C-SRR-GR hybrid metamaterials employing a two-piece C-SRR design and residing on a SiO_2_/Si substrate.

The proposed hybrid metamaterial concept can be applied to different types of structures, but in this work the specific realization is based on GRs and electric-field-coupled complementary split-ring resonators (C-SRRs)^38^,^39^,^40^. The schematics of a C-SRR unit cell, a GR and a unit cell of the proposed hybrid structure are illustrated in Fig. 1a–c. The rationale for such a hybrid structure design is the following: the near-field electric field (E-field) distribution associated with the LC-resonance of the C-SRR is highly localized and enhanced within the capacitor gap as shown by the simulation in Fig. 1d (see Methods), while the E-field distribution of the GR localized SP resonance is also highly confined in the vicinity of the GR (Fig. 1e). Both resonances are excited by E-field in the *x* direction^26^,^38^, and upon excitation their highly confined near-field also has the dominant E-field component in the *x* direction. Therefore, embedding the GR in the middle of the C-SRR capacitor gap is an effective way to achieve strong near-field coupling of the two structures, and the resulted hybrid structure forms a coupled oscillator system. When the localized SP resonance of the GR is tuned to approach and subsequently surpass the C-SRR LC-resonance by electrostatically varying the carrier density (*ω*_SP_∝*n*^1/4^∝|*E*_F_|^1/2^, where *ω*_SP_ is the frequency of the SP resonance, *n* is the carrier density and *E*_F_ is the Fermi energy), the spectral response of the hybrid metamaterial is modulated in the frequency range containing the two resonances. Such a mechanism of transmission modulation is fundamentally different from those exploiting free-carrier absorption^12^,^13^,^41^,^42^,^43^, which introduces tunable damping to the resonance. In addition to efficient transmission modulation, the strong coupling between the two resonators also lead to further near-field localization and enhancement in comparison with either resonator alone; whereas, in previously demonstrated tunable metamaterials employing resonators on a continuous graphene layer^41^,^42^,^43^, the near-field enhancement of the resonators is reduced due to significant damping by the free carriers. Owing to the confined dimension along the GR width, the Drude-like free-carrier absorption is suppressed when the incident radiation is polarized perpendicular to the GR, and the localized SP resonance is the dominant process^26^. To achieve large modulation, the C-SRR capacitor gap should be designed to accommodate the GR with small margins to maximize the field overlap and hence the coupling strength. In addition, precise control of the hybrid metamaterial spectral response requires accurate information on the charge neutrality point (CNP) of the GRs. Therefore, a modified unit cell design is developed in which a narrow gap along the horizontal symmetry axis is introduced to separate the C-SRR into two parts (see Fig. 1f and Supplementary Note 1, Supplementary Fig. 1) with minimal influence on its spectral response and field distribution. This slight structural variation allows for convenient electrical characterization of the GR (such as the CNP and carrier mobility) and current annealing with the two C-SRR parts functioning as separate contacts, and may also lead to other applications, such as direct probing of the GR photo-response^35^,^36^ as influenced by the C-SRR cavity, or passing a current through the GR to exploit antenna (C-SRR) enhanced thermal radiation from the GR plasmonic resonators^44^.

### Device fabrication

**Figure 2:**
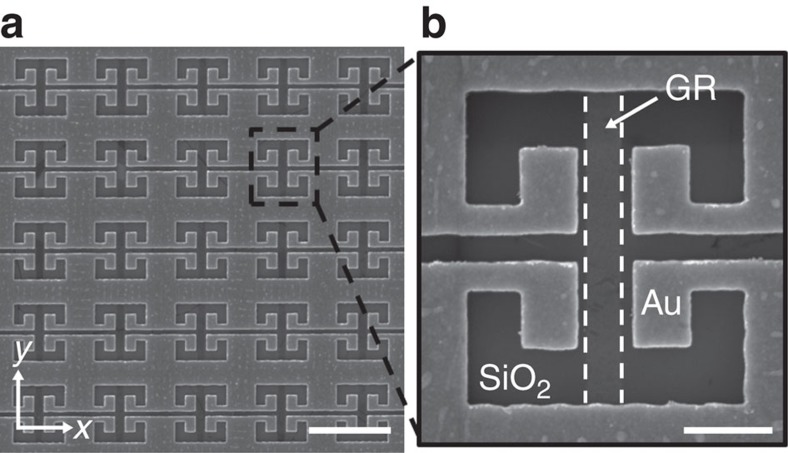
SEM images of a fabricated C-SRR-GR hybrid metamaterial device (HM1). (**a**) SEM image of an array of C-SRR-GR unit cells. Scale bar, 5 μm. (**b**) Close-up SEM image of a single C-SRR-GR unit cell. Scale bar, 1 μm.

Following the above design principle, we have developed and experimentally investigated multiple different hybrid metamaterial structures targeting two different operating frequency ranges, that is, ∼10 and ∼4.5 THz. To achieve this, GR widths of ∼400 nm and ∼1.8 μm, respectively, are chosen to ensure that the SP resonance can be electrostatically tuned across the C-SRR LC resonance^31^, even in the presence of moderate screening effect from the surrounding metal (see Supplementary Note 2, Supplementary Fig. 2). To achieve a large coupling strength, the C-SRR capacitor gap is designed to be wider than the enclosed GR by 200–260 nm for the ∼10 THz structures, and wider by 200–600 nm for the ∼4.5 THz structures, respectively. The GRs are implemented with large-area monolayer graphene grown by chemical vapour deposition (CVD) and transferred onto a SiO_2_/Si substrate, which is also utilized as the back-gate for electrostatic control of the graphene carrier density. Electron-beam lithography is employed for patterning the structures (see Methods). Figure 1f shows a three-dimensional schematic representation of the final device, and Fig. 2 shows scanning electron microscope (SEM) images of a fabricated C-SRR-GR array designed to operate around 10 THz.

### Strong coupling-induced transmission modulation

**Figure 3:**
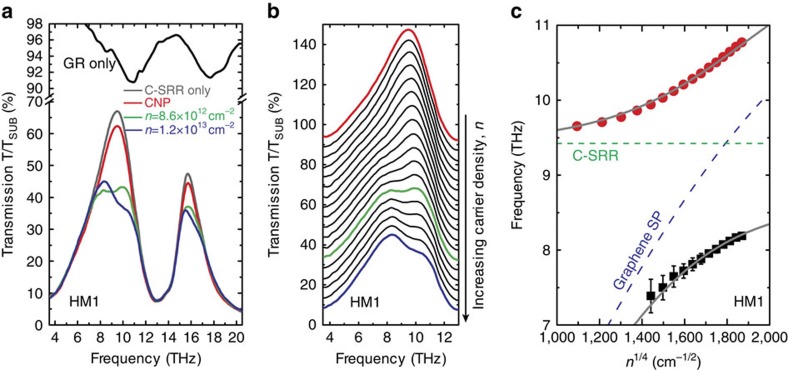
Transmission modulation of C-SRR-GR hybrid metamaterials operating at ∼10 THz. (**a**) Transmission spectra (normalized to the substrate transmission) of the C-SRR-GR hybrid metamaterial HM1 at different graphene carrier densities in comparison with the transmission spectrum of a reference bare C-SRR array. The transmission spectrum of a GR array exhibiting the localized SP resonances is also shown. (**b**) Stacked transmission spectra of HM1 (5% offset) at varied carrier densities (from the CNP to ∼1.2 × 10^13^ cm^−2^). The coloured curves in **b** correspond to those in **a** of the same colour. (**c**) Symbols are the extracted peak frequencies of the two hybridized modes in the corresponding transmission spectra in **b**, plotted as a function of the carrier density (*n*^1/4^). The choice of *n*^1/4^ as the *x*-axis variable in **c** is because of the fact that the resonance frequency of uncoupled graphene surface plasmon is proportional to *n*^1/4^, which is different from the *n*^1/2^ dependence associated with two dimensional electron gas in semiconductor heterostructures^26^. The solid curves are fits of the data points with equation (1) describing the anti-crossing of two strongly coupled resonances, that is, the localized SP resonances in the GRs (blue dashed line) and the C-SRR LC-resonance (green dashed line). See Supplementary Note 3 for more details of the fitting procedure.

To investigate the carrier density-dependent spectral response of the fabricated C-SRR-GR hybrid metamaterial devices, their transmission spectra are characterized employing Fourier transform infrared spectroscopy (FTIR) with the normally incident radiation polarized perpendicular to the GRs (see Methods). Figure 3 summarizes the key results from a hybrid metamaterial device (HM1) designed to operate around 10 THz. Figure 3a shows the transmission spectra of HM1 at three different carrier densities in comparison with that of a reference bare C-SRR array (the transmission of the SiO_2_/Si substrate is ∼45% in this frequency range, and its reflection is estimated to be ∼30%). The bare C-SRR array exhibits two resonances in the measured frequency range due to the presence of an optical phonon mode of the underlying SiO_2_ layer^45^. Figure 3a also shows the transmission spectrum of an array of 400-nm-wide GRs with a carrier density of 2.3 × 10^13^ cm^−2^, in which two resonances originating from the hybridization of the intrinsic graphene SP resonance and the SiO_2_ optical phonon mode are present^31^ (see Supplementary Note 3, Supplementary Fig. 3). The quality factors (Q) of all these resonances are larger than 3, in part because of coupling to the SiO_2_ optical phonon. Both graphene SP resonances interact with the corresponding C-SRR resonances with similar frequency; however, the interaction between the lower frequency ones (∼10 THz) are more effective, thanks to the relatively stronger resonances and better frequency matching at high graphene carrier density. Focusing on the spectral range near the lower frequency LC-resonance, it is evident that at the CNP, the transmission spectrum of the device is close to that of the corresponding reference C-SRR array, whereas large transmission modulation in both amplitude and line shape is realized by varying the carrier density, with more than 40% relative modulation of the peak transmission achieved with HM1. This is in sharp contrast to previous investigations where a continuous layer of graphene is brought into contact with SRRs^41^,^42^ and the LC-resonance of the SRRs is considerably diminished even at the nominal CNP condition^42^, as both the inductive and the capacitive components in the equivalent LC-circuit of a SRR are shunted by the free carriers in the continuous graphene layer.

Moreover, the transmission spectra at high carrier densities display a double-peak feature. This is a characteristic feature of the strong coupling^46^,^47^ between the C-SRR LC-resonance and the GR localized SP resonance due to efficient near-field interaction, which leads to two hybridized modes. The evolution of the transmission of HM1 with increasing carrier density is more clearly revealed in Fig. 3b, in which the higher frequency peak is observed to gradually blue-shift with decreasing strength, while the lower frequency peak emerges and becomes more pronounced at higher carrier densities. The frequencies of the individual peaks in the transmission spectra are extracted by fitting the data with two Lorentzian functions (since the resonances are relatively broad, the expected slight asymmetry in the line shape does not significantly affect the quality of the fitting; other fitting functions such as Gaussians produce very similar results on the fit peak positions), and the results are plotted in Fig. 3c. The dispersions of the upper and the lower branches of the hybridized modes are well fitted with a formula


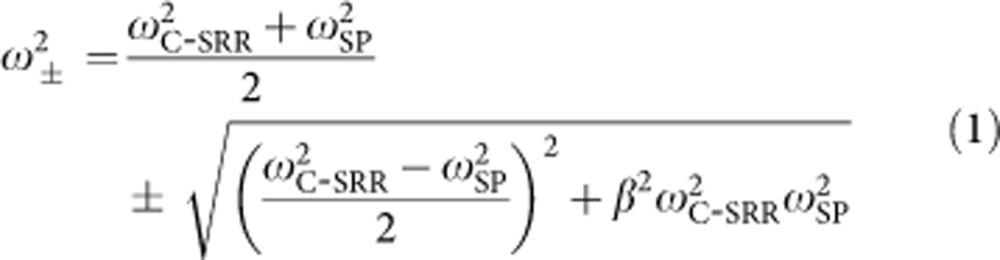


describing the anti-crossing behaviour of two strongly coupled resonances^48^, where *β* is a parameter determining the coupling strength, and may also be interpreted with an equivalent circuit model as the relative strength of a capacitive coupling between two LC-resonators (see Supplementary Note 4, Supplementary Fig. 4 and 5). The splitting between the two branches (*ω*_+_– *ω*_−_) when *ω*_SP_=*ω*_C−SRR_ is approximately *βω*_C−SRR_, and the coupling strength (half of this splitting) is found to be ∼1.2 THz in both devices, more than 10% of the individual resonance frequency and is therefore also within the ultrastrong coupling regime^49^. The response of such a system consisting of strongly coupled subwavelength resonators may also be described under the framework of Fano resonance^50^.

### Comparison with numerical simulations

**Figure 4:**
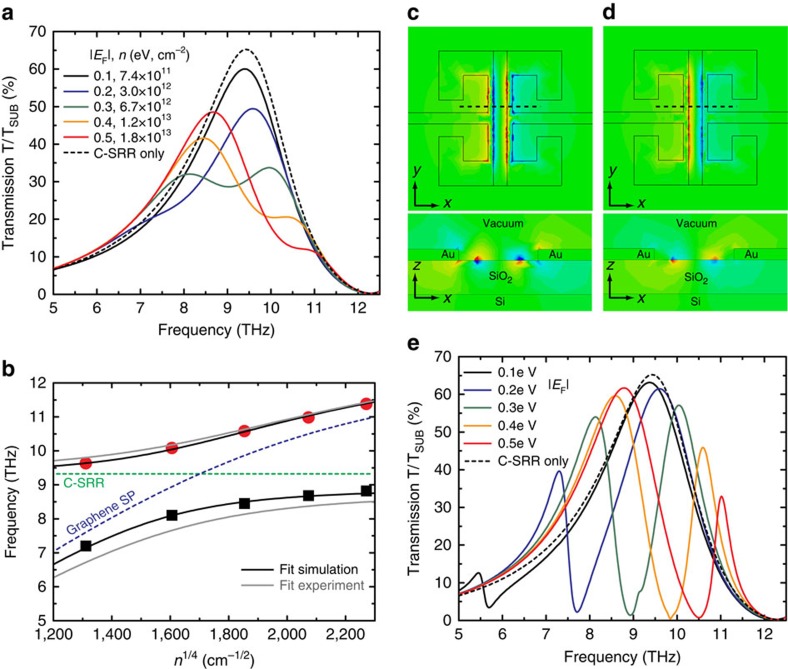
Simulated spectral response of C-SRR-GR hybrid metamaterials operating at ∼10 THz. (**a**) Simulated transmission spectra of the C-SRR-GR hybrid metamaterial HM1 at various graphene Fermi energies (carrier densities) in comparison with that of a reference bare C-SRR array. The carrier relaxation time of 50 fs is used in this simulation. (**b**) Symbols are the extracted peak frequencies of the two hybridized modes in **a** as a function of the graphene carrier density (*n*^1/4^). The solid black curves are fits of the data points using equation (1), with the dashed lines of the same meaning as in Fig. 3c. The fits of the corresponding experimental data shown in Fig. 3c are also plotted in solid grey curves for comparison. (**c**,**d**) Simulated *z*-component of the E-field distributions associated with the two hybridized modes (transmission peaks), that is, the bonding mode (**c**) and the anti-bonding mode (**d**), at |*E*_F_|=0.3 eV. The upper graphs correspond to the field distributions in the *x*–*y*-plane right below the C-SRR-GR unit cell, and the lower graphs correspond to those in the *x*–*z*-plane indicated by the dashed line in the upper graphs. (**e**) Simulated carrier-density-dependent transmission spectra of a C-SRR-GR hybrid metamaterial in comparison with that of a reference bare C-SRR array, assuming 1 ps for the graphene carrier relaxation time.

These experimental observations are well reproduced by full-wave simulations. Figure 4a shows the simulated transmission spectra of HM1 at various carrier densities in comparison with that of a bare C-SRR array. Both the large transmission modulation and the double-peak profile at high carrier densities are consistent with the experimental observations. The two hybridized modes exhibit a typical anti-crossing behaviour (Fig. 4b) as well as exchange of their oscillator strengths, as the localized SP resonance approaches and subsequently surpasses the LC-resonance with increasing carrier density. The coupling strength is extracted to be ∼1.0 THz (see Supplementary Note 3, Supplementary Table 1), in good agreement with the experimental result (∼1.2 THz). The simulated E-field profiles associated with the two transmission peaks at |*E*_F_|=0.3 eV (Fig. 4c–d) evidently reveal that the lower frequency peak stems from a bonding mode, and the higher frequency peak an anti-bonding mode^51^. As an additional benefit of the strong coupling, the field localization and enhancement near the GR in the hybrid structure is considerably increased compared with either the bare C-SRR or the bare GR (for example, the field enhancement near the GR edges in the hybrid structure is approximately one order of magnitude higher than that in the bare GR array at its SP resonance, as shown in Supplementary Note 5, Supplementary Fig. 6), which leads to enhanced absorption of incident radiation in the GR, and may also find broad applications in chemical and biological sensing. Furthermore, with graphene of higher material quality and thus higher carrier mobility, the carrier density-dependent modulation of the transmission is expected to be further improved. As shown in the simulated spectra in Fig. 4e assuming a carrier mobility that is realistic for the state-of-the-art CVD grown graphene^52^,^53^, the transmission of such a hybrid metamaterial can be switched almost completely off across a wide frequency range by controlling the graphene carrier density. Such a superior modulation performance is another direct consequence of the strong coupling between the two resonances.

### High-speed modulation of THz radiation

**Figure 5:**
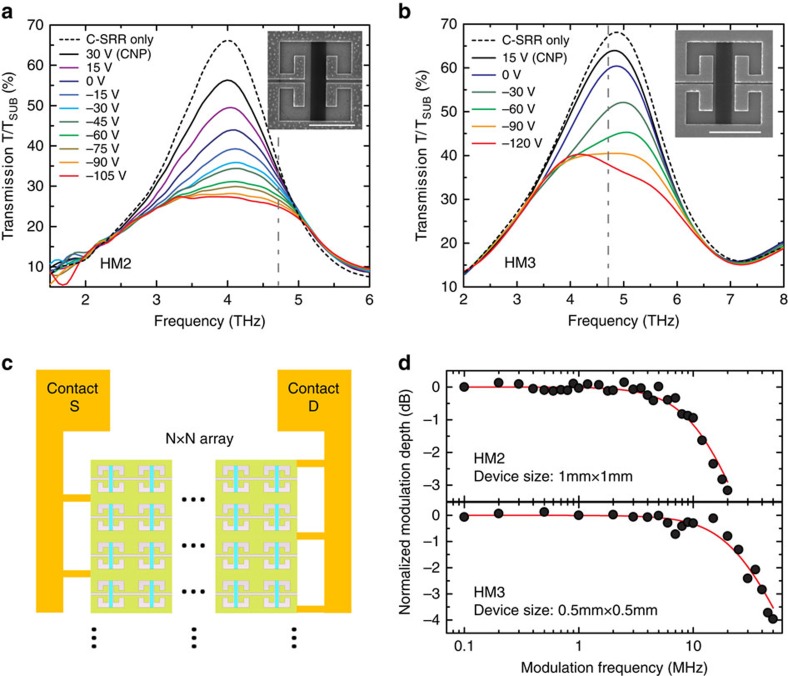
Transmission modulation of C-SRR-GR hybrid metamaterials operating around 4.5 THz. (**a**,**b**) Transmission spectra (normalized to the substrate transmission) of the C-SRR-GR hybrid metamaterial devices HM2 (**a**) and HM3 (**b**) at different back-gate voltages (graphene carrier densities) in comparison with the transmission spectrum of the corresponding reference bare C-SRR array. The inset in each graph shows a SEM image of a device unit cell. Scale bars, 5 μm. The vertical grey dashed lines indicate the frequency of the THz quantum cascade laser used in the modulation speed measurement. (**c**) Schematic representation of the parallel electrical contact for all the C-SRR-GR unit cells. (**d**) Symbols are the normalized modulation depth of two hybrid metamaterial devices, HM2 (top) and HM3 (bottom), as a function of modulation frequency. Solid curves are the fits of the measured data based on a standard RC circuit model.

The demonstrated C-SRR-GR hybrid metamaterials can be utilized as efficient modulators and switches. Since fast modulation is highly desired for many applications such as real-time compressive imaging^11^,^54^ and wireless communication, the modulation speed of several devices operating around 4.5 THz are investigated using a THz quantum cascade laser (4.7 THz) as the source. Figure 5a–b show the transmission spectra of two hybrid metamaterial structures (HM2 and HM3) operating at ∼4.0 and ∼4.8 THz, respectively, at various back-gate voltages in comparison with the transmission spectrum of the corresponding reference bare C-SRR array. The transmission exhibits similar (even higher) carrier density-dependent modulation as observed in the 10 THz devices, with ∼60% relative modulation of the peak transmission. The plateau-shaped transmission spectra in both figures correspond to the situation where the two hybridized modes have similar strengths, and they also suggest that the two resonances are in the critical coupling regime, that is, the coupling strength is close to half of the broadening, and thus the individual transmission peaks associated with each hybridized mode are not as clearly resolved as in the 10 THz devices, consistent with the simulation (see Supplementary Note 6, Supplementary Fig. 7). This is a result of the relatively lower quality factor of both the GR SP resonance and the C-SRR LC-resonance in this frequency range (Q∼2). The C-SRR-GR structure allows for straightforward parallel electrical connection of all the unit cells, as illustrated in Fig. 5c, to minimize the total resistance of the device. Hence, as the total capacitance (*C*) increases proportional to the device area, the total resistance (*R*) associated with the entire C-SRR-GR array scales inversely proportional to the device area, facilitating high-speed operation of large-area devices. Figure 5d shows the modulation speed measurement (see Methods) of HM2 (1 mm × 1 mm area) and HM3 (0.5 mm × 0.5 mm area), respectively. The measured 3 dB cut-off frequency is ∼19 MHz for the larger device and ∼41 MHz for the smaller device. To the best of our knowledge, such performance is superior to the state-of-the-art for fast tunable metamaterials in the literature, which was achieved with much smaller device area^55^, and is several times higher than that reported for devices with similar area^54^. Electrical characterizations show that the resistance of the C-SRR-GR array is indeed not the limiting factor for the RC constant of these devices, whereas the dominant resistance contribution is from the low-doped Si substrate (because of the in-plane current flow) and the input resistance (50 Ω) of the driving voltage source (see Supplementary Note 7, Supplementary Fig. 8). The modulation speed can be further enhanced up to GHz range without the need of reducing the device area. For example, by utilizing a wire–grid contact on the backside of the Si substrate, the substrate contribution to the total resistance can be reduced by more than one order of magnitude without affecting the transmission of THz radiation. The total capacitance can also be significantly reduced with further optimization of the device architecture, such as employing a transparent local top-gate for only the GRs or using resonator structures covering less area.

The demonstrated concept of coupling graphene-based plasmonic structures with conventional metal-based metamaterials to achieve highly tunable hybrid metamaterials can be straightforwardly extended to other frequency ranges (for example, mid-infrared) and different graphene plasmonic and/or metameterial structures (for example, graphene disks and negative-index metamaterials), to further broaden the scope of novel functionalities. The presented device structures also provide a platform for further exploring intriguing cavity-enhanced light–matter interactions (for example, cavity quantum electrodynamics in the ultrastrong coupling regime^56^) and optical processes in graphene plasmonic structures, which may lead to various additional applications such as sensing, photo-detection, enhanced thermal emission and nonlinear frequency generation.

## Methods

### Device fabrication

The C-SRR-GR hybrid metamaterials are fabricated from pre-transferred large-area CVD grown monolayer graphene on 300-nm thick SiO_2_, which has an underlying Si substrate with resistivity of ∼10 Ωcm. Electron-beam lithography and reactive ion etching with O_2_ plasma are used to pattern the continuous graphene sheet into arrays of ribbons with the designed widths and separations. A second stage of electron-beam lithography followed by deposition of Cr/Au (thickness 5/80 nm) and a lift-off process defines the C-SRR arrays, which are in direct contact with the GRs to enable electrostatic control of the carrier density using the Si substrate as the back-gate, as well as direct characterization of the electrical properties of the GRs. The devices operating around 10 THz have a surface dimension of 2 mm × 2 mm or 0.6 mm × 0.6 mm, and the devices operating around 4.5 THz have various surface dimensions: 2 mm × 2 mm; 1 mm × 1 mm or 0.5 mm × 0.5 mm.

### Transmission characterization

Transmission spectra of the C-SRR-GR hybrid metamaterial devices are characterized with FTIR spectroscopy. The measurements are performed at room temperature in the vacuum chamber of a Bruker Vertex 80v FTIR, with the normally incident radiation linearly polarized perpendicular to the GRs using a wire–grid polarizer. The resolution is 8 cm^−1^ for the ∼10 THz devices and 2 cm^−1^ for the ∼4.5 THz devices. The low-frequency measurement range is limited to ∼1 THz by the DTGS detector, while the high-frequency measurement range is significantly above the operating frequencies of the investigated devices.

### Modulation speed measurement

The modulation speed measurement is conducted with a 4.7 THz quantum cascade laser as the light source, a function generator as the voltage source for modulating the hybrid metamaterial devices (up to 80 MHz), and a superconducting hot electron bolometer as the fast detector (with response up to 200 MHz), feeding the output signal to a lock-in amplifier with demodulation frequency up to 50 MHz (limiting the frequency range of the measurement). The output voltage from the function generator is set to be a sinusoidal signal with 10 V amplitude (limited by the equipment). The modulation depth at any specific frequency in Fig. 5d is normalized to the value measured at 1 MHz modulation frequency.

### Full-wave simulation

The spectral responses and field distributions of all the investigated structures are simulated using finite-element frequency domain methods with CST Microwave Studio. The monolayer graphene sheet is modelled as a 0.3 nm thin layer with a dynamical surface conductivity described by the Drude model as 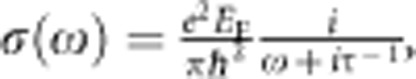, which is an accurate approximation when the Fermi energy *E*_F_ is significantly higher than the corresponding energy of the frequency range investigated. In most of the simulations, the carrier relaxation time is assumed to be 50 fs which is realistic for the graphene material available for our experimental demonstration^31^. The frequency-dependent permittivity of the SiO_2_ layer (300 nm thick) is computed taking into account the surface optical phonon mode at ∼14.5 THz (see Supplementary Note 3), while tabulated data from ref. 57 are used for the low-doped Si substrate (modelled as 5 μm thick). The C-SRR structure is modelled to consist of 100-nm thick gold, with the permittivity described by the Drude model assuming the plasma frequency of 2*π* × 2.184 × 10^15^ s^−1^ and the damping constant of 2*π* × 1.7 × 10^13^ s^−1^.

## 

## Additional information

**How to cite this article:** Liu, P. Q. *et al*. Highly tunable hybrid metamaterials employing split-ring resonators strongly coupled to graphene surface plasmons. *Nat. Commun.* 6:8969 doi: 10.1038/ncomms9969 (2015).

## Supplementary Material

Supplementary Information Supplementary Figures 1-8, Supplementary Table 1, Supplementary Notes 1-7 and Supplementary References.
