# Supplementary material for: Highly tunable hybrid metamaterials employing split-ring resonators strongly coupled to graphene surface plasmons
Source: Nat Commun. 2015 Nov 20;6:8969. doi: 10.1038/ncomms9969 (PMC4673875; doi:10.1038/ncomms9969)
Supplement: Supplementary Information — Supplementary Figures 1-8, Supplementary Table 1, Supplementary Notes 1-7 and Supplementary References. [file ncomms9969-s1.pdf]

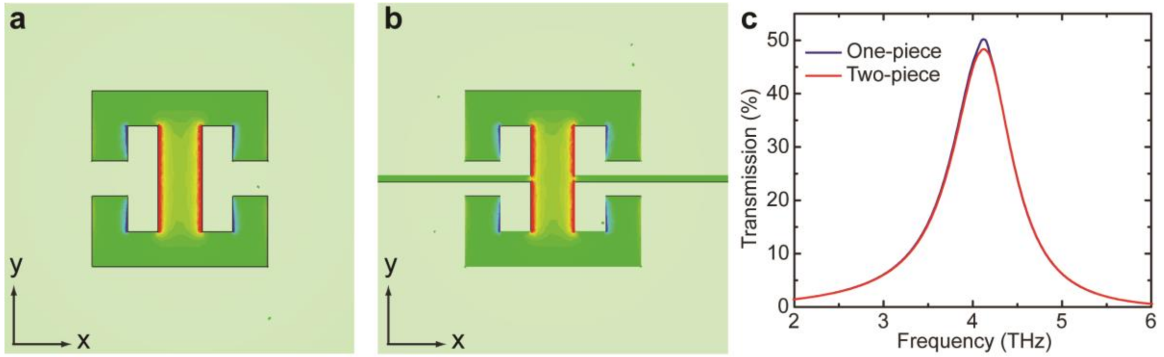

Supplementary Figure 1 | Simulated E-field distributions and transmission spectra of two different C-SRR structures. **a,** Simulated *x*-component of the near-field E-field distribution of a conventional (one-piece) C-SRR design. **b,** Simulated *x*-component of the near-field E-field distribution of a two-piece C-SRR design. **c,** Comparison of the transmission spectra of the two C-SRR designs in (**a**) and (**b**).

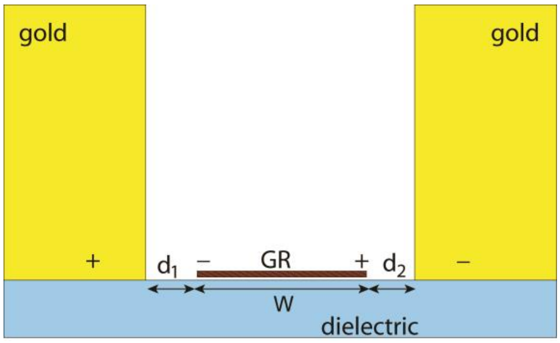

Supplementary Figure 2 | Schematic of a GR placed between two relatively thick metallic (gold) structures with narrow gaps. Induced image charges are represented by the positive (+) and negative (-) signs in the metallic structures.

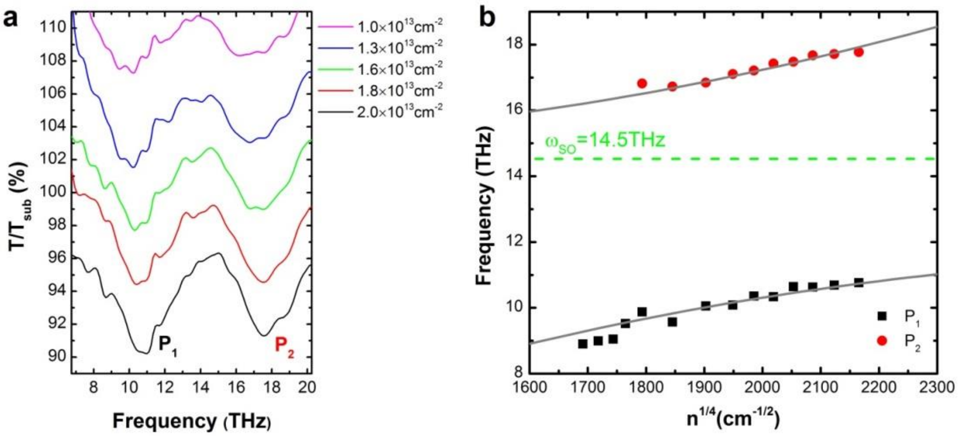

Supplementary Figure 3 | Experimental investigation of a reference array of bare GRs. **a,** Transmission spectra of an array of GRs (400 nm wide) normalised to the substrate transmission for different values of the carrier density. The labels P_1 and P_2 indicate the two SP3 modes. The curves are offset by 2%. **b,** Extracted peak

frequencies for P_1 and P_2 plotted as a function of $n^{1/4}$. The solid grey lines show a fit to the experimental data with Eq. 12.

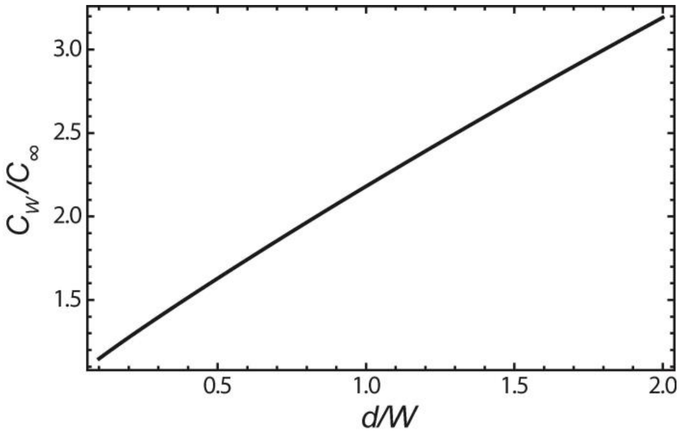

Supplementary Figure 4 | The calculated ratio C_W/C_∞ as a function of d/W at $\epsilon = 3.9$.

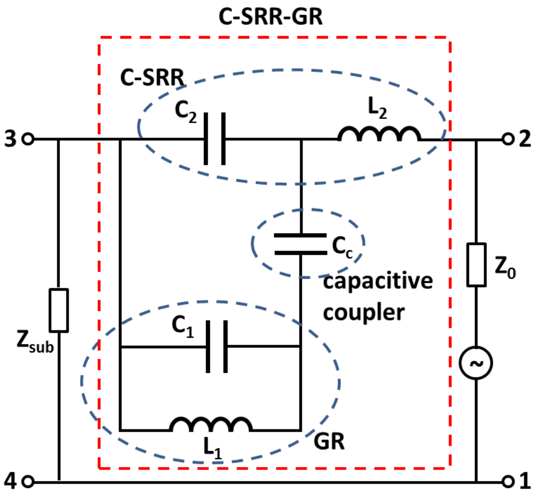

Supplementary Figure 5 | Equivalent circuit diagram of the C-SRR-GR hybrid metamaterial.

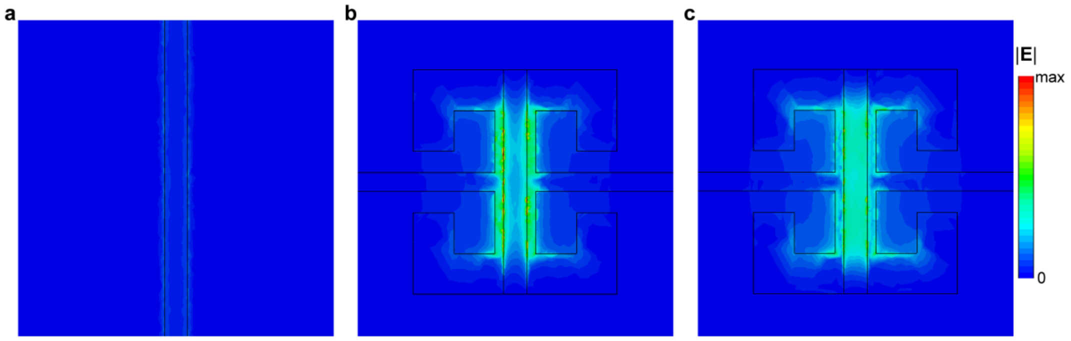

Supplementary Figure 6 | Comparison of the simulated E-field amplitude distributions between GR and C-SRR-GR. a, Simulated E-field amplitude distribution of the plasmonic resonance of a GR at E_F=0.3 eV. **b,c,** Simulated E-field amplitude distributions of the bonding mode (**b**) and the anti-bonding mode (**c**) of a C-SRR-GR hybrid structure HM1 at E_F=0.3 eV (near the anti-crossing point).

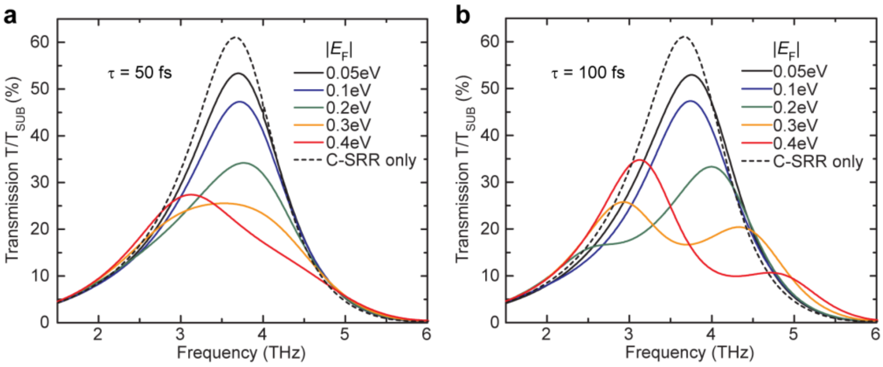

Supplementary Figure 7 | Simulated transmission spectra of tunable C-SRR-GR hybrid metamaterials operating at ~4 THz. a,b, Simulated transmission spectra of device HM2 at various graphene Fermi energies ($|E_F|$) in comparison with that of the reference bare C-SRR array, assuming two different carrier relaxation time τ, i.e. $\tau = 50$ fs (**a**) and $\tau = 100$ fs (**b**), respectively.

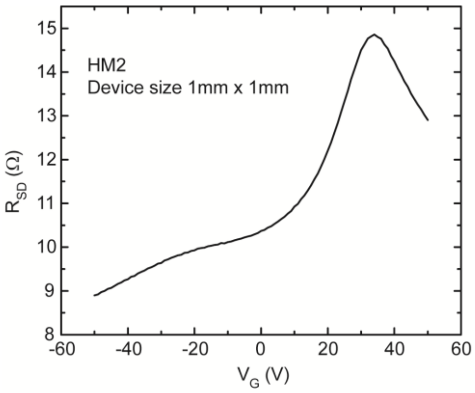

Supplementary Figure 8 | Measured source-drain resistance (R_{SD}) of the entire C-SRR-GR array (HM2) as a function of the back-gate voltage (V_G).

Supplementary Table 1 | C-SRR-GR fit parameters.

	Experiment (HM1)	Simulation (HM1)
A	0.74	0.82
β	0.25	0.21
$\omega_{C\text{-}SRR}$ (THz)	9.3	9.3

Supplementary Note 1. Comparison between a conventional C-SRR and a modified two-piece C-SRR designs

By introducing a narrow gap along the horizontal symmetry axis of a conventional C-SRR unit cell, the C-SRR is electrically separated into two parts with minimal influence on the near-field E-field distribution (as indicated by the comparison between Supplementary Fig. 1a and Supplementary Fig. 1b) and the transmission spectrum (shown in Supplementary Fig. 1c).

Supplementary Note 2. Screening of the GR localized plasmons by nearby metallic structures

If the distance between the edges of a GR and the nearby metallic structures (such as the C-SRR in this work) is sufficiently small compared to the thickness of the metallic structures, the localized SP resonance frequency of the GR is reduced as a result of the screening effect from the metal. The collective oscillation of the charge carriers (i.e. the SP resonance) in a GR is a consequence of the restoring force

$$F_x(x) \propto \frac{eQ_l}{\epsilon}\left(\frac{1}{|x+W/2|}+\frac{1}{|x-W/2|}\right), \quad |x|<\frac{W}{2}, \tag{1}$$

arising due to the charge density fluctuations with the linear charge density Q_l, localized near the GR edges and indicated by the positive (+) and negative (-) signs in Supplementary Fig. 2. In the presence of nearby thick (compared to the distances d_1 and d_2) metallic structures which may be approximated as infinitely high metallic walls extending in both directions, this restoring force is modified by the image charges induced in the metal,

$$F_x(x) \propto \frac{eQ_l}{\epsilon}\left(\frac{1}{|x+W/2|}+\frac{1}{|x-W/2|}-\frac{1}{|x+W/2+2d_1|}-\frac{1}{|x-W/2-2d_2|}\right), \quad |x|<\frac{W}{2}. \tag{2}$$

As a result, the SP resonance frequency is reduced to

$$\omega'_{\mathrm{SP}} = \omega^0_{\mathrm{SP}}\sqrt{1-\frac{1}{2(1+\alpha_1)}-\frac{1}{2(1+\alpha_2)}}, \tag{3}$$

where ω^0_{SP} is the unscreened SP resonance frequency of the GR and $\alpha_{1,2} = 4d_{1,2}/W$. This calculation provides the theoretical upper limit of the screening effect induced by nearby metallic structures, while the actual reduction of the SP resonance frequency depends on the geometrical details of the structures and can be computed numerically.

Supplementary Note 3. Fitting procedure of the hybridized modes of the C-SRR-GR hybrid metamaterials

The efficient coupling of the C-SRR LC resonance to the GR localized SP resonance leads to the observed hybridized modes in the transmission spectra of the C-SRR-GR hybrid metamaterials. To quantitatively analyse the carrier density dependent spectral response of these hybrid metamaterials, we have also investigated the tuning of the SP resonances in bare GR arrays (without the C-SRRs). The GR arrays are fabricated on the same SiO_2/Si substrates used for the hybrid metamaterials, which allow for tuning of the carrier density via the applied back-gate voltage [1]. SiO_2 is a polar dielectric and as such, supports surface optical (SO) phonons, which can couple to SP resonances in the GRs via the long-range Fröhlich interaction [2,3], resulting in multiple surface-plasmon-phonon-polariton (SP3) modes [1,4]. The transmission spectrum of such an array of 400-nm-wide GRs is measured as a function of carrier density and plotted in Supplementary Fig. 3a. In the measured frequency range, two resonances are observed, labelled P_1 and P_2, which originate from the interaction of the graphene SP and the SO phonon at ~14.5 THz [1]. As the carrier density is increased, there is a monotonic increase in the frequency of both resonances, with the peak positions plotted as a function of carrier density ($n^{1/4}$) in Supplementary Fig. 3b.

To derive an equation to describe the anti-crossing of the plasmon and SO phonon modes, we consider a 2D layer with the conductivity $\sigma(\omega)$ lying on the boundary $z = 0$ of two media with the bulk dielectric functions $\epsilon_1(\omega)$ and $\epsilon_2(\omega)$. The spectrum of electromagnetic waves running along this boundary in the x-direction and localized at this boundary is determined by the dispersion equation (in Gaussian units)

$$\varepsilon_1(\omega) + \varepsilon_2(\omega) + \frac{4\pi i|q|\sigma(\omega)}{\omega} = 0\,. \tag{4}$$

The conductivity can be described using the Drude model, which for the case of graphene and neglecting the carrier relaxation is

$$\sigma(\omega) = \frac{e^2 v_{\mathrm{F}}}{\hbar}\sqrt{\frac{n}{\pi}}\frac{i}{\omega}, \tag{5}$$

where n is the sheet carrier density and v_{F} is the Fermi velocity. We assume the first medium is vacuum, $\epsilon_1(\omega) = 1$ and the second has the dielectric function $\epsilon_2(\omega)$ and consider several special cases. Firstly, the second medium is also vacuum $\epsilon_2(\omega) = 1$. The dispersion equation then gives

$$\omega^2 = \frac{2\pi|q|e^2 v_{\mathrm{F}}}{\hbar}\sqrt{\frac{n}{\pi}} \equiv \omega_{\mathrm{p}}^2\,, \tag{6}$$

where we define ω_{p} as the plasma frequency for graphene in vacuum. In the second case, we assume there is no graphene, i.e. $n = 0$, and the second medium is a polar semiconductor with

$$\epsilon_2(\omega) = \epsilon_\infty - (\epsilon_0 - \epsilon_\infty)\frac{\omega_{\mathrm{TO}}^2}{\omega^2 - \omega_{\mathrm{TO}}^2}, \tag{7}$$

where ω_{TO} is the frequency of the transverse optical phonons, and ϵ_0 and ϵ_∞ are the dielectric constants at frequencies much lower and much higher than ω_{TO}, respectively. The dispersion equation gives

$$1 + \epsilon_2(\omega) = 1 + \epsilon_\infty - (\epsilon_0 - \epsilon_\infty)\frac{\omega_{\mathrm{TO}}^2}{\omega^2 - \omega_{\mathrm{TO}}^2} = 0\,, \tag{8}$$

and can be rewritten as

$$\omega^2 = \omega_{\mathrm{SO}}^2 = \omega_{\mathrm{TO}}^2\frac{\epsilon_0 + 1}{\epsilon_\infty + 1}, \tag{9}$$

where ω_{SO}^2 is the frequency of the SO phonons. Therefore, the dispersion equation at the interface of a vacuum and a polar semiconductor can be written as

$$1+\epsilon_2(\omega)=(1+\epsilon_\infty)\frac{\omega^2-\omega_{\mathrm{SO}}^2}{\omega^2-\omega_{\mathrm{TO}}^2}=0\,.$$

(10)

Finally, for the case of graphene on the surface of a polar crystal, the dispersion equation is

$$1+\epsilon_2(\omega)-2\frac{\omega_{\mathrm{p}}^2}{\omega^2}=(1+\epsilon_\infty)\frac{\omega^2-\omega_{\mathrm{SO}}^2}{\omega^2-\omega_{\mathrm{TO}}^2}-2\frac{\omega_{\mathrm{p}}^2}{\omega^2}=0\,,$$

(11)

which leads to the following biquadratic equation, where the two solutions describe the coupled plasmon-phonon modes,

$$\omega_{\mathrm{SP},\pm}^2=\frac{\omega_{\mathrm{SO}}^2+\frac{2\omega_{\mathrm{p}}^2}{\epsilon_\infty+1}}{2}\pm\frac{\sqrt{\left(\omega_{\mathrm{SO}}^2-\frac{2\omega_{\mathrm{p}}^2}{\epsilon_\infty+1}\right)^2+8\omega_{\mathrm{SO}}^2\omega_{\mathrm{p}}^2\left(\frac{1}{\epsilon_\infty+1}-\frac{1}{\epsilon_0+1}\right)}}{2}\,.$$

(12)

To fit the experimental data shown in Supplementary Fig. 3b, we use Eq. 12 with ω_{p} defined according to Eq. 6 with a slight modification as

$$\omega_{\mathrm{p}}=\sqrt{\frac{2\pi|q|e^2v_{\mathrm{F}}}{\hbar\sqrt{\pi}}}n^{1/4}\rightarrow A\sqrt{\frac{2\pi^2e^2v_{\mathrm{F}}}{\sqrt{\pi}\hbar W}}n^{1/4},$$

(13)

where A is a fitting parameter to take into account the relation between the frequency of the localized SP resonance in a GR and the frequency of a SP wave with the wave vector $|q|$ in a continuous graphene sheet (Eq. 6); the factor A was numerically found to be $A\sim0.86$ in [5], see Figure 1 there (see also [6]). The solid lines in Supplementary Fig. 3b show a fit to the experimental data using Eq. 12, with ω_{SO} = 14.5 THz (ref 1), ϵ_0=3.9 and the fit parameters ϵ_∞=2.81 and A=0.77. The lower branch, $\omega_{\mathrm{SP},-}$ is subsequently used to fit the anti-crossing of the C-SRR-GR device HM1, employing Eq. 1 in the main text with $\omega_{\mathrm{SP}}=\omega_{\mathrm{SP},-}$, and with ω_{SO} = 14.5 THz, ϵ_0=3.9, ϵ_∞=2.81 as fixed parameters and A, β, $\omega_{\mathrm{C\text{-}SRR}}$ as fitting parameters. The same fitting procedure is carried out for the simulated transmission spectra of HM1 in Fig. 4 of the main text. Supplementary Table 1 lists the fit parameters of both the experimental and the simulated transmission spectra of HM1. Excellent agreement is found between the experimental results and the simulation.

It is worth pointing out that when evaluating the experimental carrier density of the 400 nm wide GRs at a given back-gate voltage, the parallel-plate capacitor model is not a good approximation in this case, as the GR width is comparable to the thickness d (~300 nm) of the dielectric layer and the edge effect is hence significant. To this end we consider a thin conducting layer of the width W (the GR) lying on a dielectric slab at a distance d from the grounded conducting substrate. The distribution of the electric potential ϕ in the free space and in the dielectric is described by the Laplace equation with the boundary conditions $\phi=0$ at the grounded substrate and $\phi=V_{\mathrm{G}}$ at the GR. Solving this equation with the Green function technique, we reduce the problem to an integral equation for the electron density $n(x)$ inside the GR

$$V_{\mathrm{G}} = \int_{-\infty}^{\infty} \left(\frac{2e}{q(1+\epsilon \coth qd)} \int_{-W/2}^{W/2} n(x') \cos(q_x x') dx' \right) \cos(q_x x)\, dq_x\,, \quad |x| < \frac{W}{2}, \tag{14}$$

where $q = |q_x|$. Expanding the density $n(x)$ in a series of Chebyshev polynomials $T_{\mathrm{n}}(2x/W)$ we reduce the problem to an infinite set of algebraic equations for the expansion coefficients. Truncating this set (the convergence is checked and found to be very fast) we get the relation between the average density of electrons in the GR $n = \langle n(x) \rangle$ and the gate voltage. The resulting ratio of the capacitances C_W/C_∞ as a function of d/W at $\epsilon = 3.9$ (SiO_2) is shown in Supplementary Fig. 4, where C_∞ is the capacitance predicted by the parallel-plate capacitor model.

Supplementary Note 4. Equivalent circuit model of the C-SRR-GR hybrid metamaterials

We introduce an equivalent circuit model as an alternative description of the operating mechanism of the C-SRR-GR hybrid metamaterials, which also provides a more intuitive interpretation of the coupling strength characterized by the parameter β in Eq. 1 in the main text. The equivalent circuit diagram for the C-SRR-GR hybrid structure is illustrated in Supplementary Fig. 5 (enclosed by the dashed square), in which both the C-SRR and the GR are modeled as LC-circuits (losses are neglected for simplicity). The GR is modeled as a parallel LC-circuit because the SP resonance corresponds to enhanced reflection, while the C-SRR is modeled as a series LC-circuit due to enhanced transmission at the resonance. Since the coupling between the C-SRR and the GR is established via the interaction between the near-field electric field of one structure and the charge distribution of the other and vice versa, such a coupling is modeled as a capacitive coupler C_{c} between the two LC-circuits in the equivalent circuit diagram. Notice that the effective inductor L_1 associated with the GR SP resonance is mainly contributed by the kinetic inductance of the charge carriers [7,8].

Since the C-SRR is excited mainly by the incident electro-magnetic waves whereas the GR is driven mostly by the enhanced electric field in the capacitor gap of the C-SRR, the equivalent circuit for the entire system is constructed as shown in Supplementary Fig. 5, with Z_0 and Z_{sub} representing the impedance of the free space and the substrate, respectively. Therefore, the transmission peaks correspond to the zeros of the total impedance of the C-SRR-GR equivalent circuit measured between port 2 and port 3, which is straightforwardly derived to be

$$Z_{\mathrm{total}}(\omega) = \frac{\omega^4 L_1 L_2 (C_1 C_2 + C_1 C_{\mathrm{c}} + C_2 C_{\mathrm{c}}) - \omega^2 (L_1 C_1 + L_2 C_2 + L_1 C_{\mathrm{c}} + L_2 C_{\mathrm{c}}) + 1}{-i\omega^3 L_1 (C_1 C_2 + C_1 C_{\mathrm{c}} + C_2 C_{\mathrm{c}}) + i\omega (C_2 + C_{\mathrm{c}})}. \tag{15}$$

The two zeros of $Z_{\mathrm{total}}(\omega)$ occur at

$$\omega_\pm^2 = \frac{L_1(C_1 + C_{\mathrm{c}}) + L_2(C_2 + C_{\mathrm{c}})}{2} \pm \frac{\sqrt{(L_1(C_1 + C_{\mathrm{c}}) - L_2(C_2 + C_{\mathrm{c}}))^2 + 4 L_1 L_2 C_{\mathrm{c}}^2}}{2}. \tag{16}$$

Let $L_1(C_1 + C_{\mathrm{c}}) = \omega_1^2$, $L_2(C_2 + C_{\mathrm{c}}) = \omega_2^2$, then Eq. 16 becomes

$$\omega_\pm^2 = \frac{\omega_1^2 + \omega_2^2}{2} \pm \sqrt{\left(\frac{\omega_1^2 - \omega_2^2}{2}\right)^2 + \beta^2 \omega_1^2 \omega_2^2}\,, \qquad \text{where } \beta = \frac{C_{\mathrm{c}}}{\sqrt{(C_1 + C_{\mathrm{c}})(C_2 + C_{\mathrm{c}})}}.$$

(S17)

The above Eq. 17 reproduces Eq. 1 in the main text describing the anti-crossing behavior of two strongly coupled resonances, and the physical meaning of β in this equivalent circuit model is the relative strength of the capacitive coupling.

Supplementary Note 5. Comparison of the simulated near-field E-field amplitude between the GR alone and the C-SRR-GR hybrid structure

Supplementary Fig. 6 shows the simulated E-field (all 3 orthogonal components included) amplitude distributions of the plasmonic resonance of a GR (Supplementary Fig. 6a) and of the two hybridized modes of a C-SRR-GR hybrid structure HM1 (Supplementary Fig. 6b,c) employing the same GR width. These field distributions correspond to the plane right below the GR or the C-SRR-GR structures. The 3 colour plots are in the same linear scale for direct comparison. The near-field enhancement associated with the C-SRR-GR is about one order of magnitude higher than that with the GR alone.

Supplementary Note 6. Simulated carrier density dependent spectral response of the C-SRR-GR hybrid metamaterials operating at ~4 THz

The simulated transmission spectra of the hybrid metamaterial device HM2 at various Fermi energies (hence carrier densities) and assuming two different carrier relaxation times (50 fs and 100 fs) are shown in Supplementary Fig. 7, in comparison with that of the reference bare C-SRR array. The transmission spectra in Supplementary Fig. 7a exhibit good agreement with the experimental observations in Fig. 5a in the main text. On the other hand, the transmission spectra in Supplementary Fig. 7b clearly reveal the double-peak feature owing to the two hybridized modes in the strong coupling regime, similar to the experimentally observed spectral responses of the 10 THz devices in Fig. 3 in the main text. From the spectra in Supplementary Fig. 7b, a coupling strength of ~0.8 THz is extracted for the coupling between the C-SRR LC resonance and the GR localized SP resonance associated with this particular structure.

Supplementary Note 7. Additional analysis of the modulation speed measurement on the C-SRR-GR hybrid metamaterials

The demonstrated modulation speed of the C-SRR-GR hybrid metamaterials is limited by the effective *RC* time constant, contributed by both the device itself and the external driving circuit. The total capacitance of the device mainly stems from the capacitor formed between the Si substrate back-gate and the C-SRR-GR array, with the ~300 nm SiO_2 layer as the dielectric medium. Since the C-SRR-GR array covers most of the device surface area, the associated capacitance can be estimated using the parallel plate capacitor model, which yields ~115 pF for a 1 mm×1 mm device (HM2) and ~29 pF for a 0.5 mm×0.5 mm device (HM3). The effective total resistance *R* is further extracted from the modulation speed measurement, and the extracted values are ~80 Ω for HM2 and ~180 Ω for HM3, respectively, excluding the 50 Ω input resistance of the driving voltage source. Thanks to the parallel electrical connection of all the C-SRR-GR unit cells, the resistance associated with the entire C-SRR-GR array is low (Supplementary Fig. 8) and contributes to only a small fraction of the total resistance. Hence, the major contribution to the device total resistance is from the relatively low-

doped Si substrate (resistivity ~10 Ωcm). Since electrical contacts for the current devices are made at the edges of the chip, the current flows in-plane in the Si substrate when charging and discharging the capacitor, resulting in large resistance. Such substrate resistance also depends on the area of the metamaterial field and its location on the chip. Since the measured device HM3 is located closer to the chip center than device HM2 and has smaller area, the substrate resistance seen by HM3 is higher. However, this substrate resistance contribution can be significantly decreased by more than one order of magnitude if electrical contacts are made on the back side of the thin Si substrate, which would facilitate the current to flow perpendicularly instead of in-plane. Such a back contact can be realized with a properly designed wire-grid structure, imposing minimal influence on the transmission of the THz radiation. Alternatively, one can also make use of such hybrid metamaterial structures to modulate the reflection of incident THz radiation, and in this case the Si substrate can be doped much higher.

Supplementary References

[1] Luxmoore, I. J. *et al*. Strong coupling in the far-infrared between graphene plasmons and the surface optical phonons of silicon dioxide. *ACS Photon.* **1**, 1151-1155 (2014).

[2] Wang, S. Q., Mahan, G. D. Electron scattering from surface excitations. *Phys. Rev. B* **6**, 4517-4524 (1972).

[3] Fratini, S., Guinea, F. Substrate-limited electron dynamics in graphene. *Phys. Rev. B* **77**, 195415 (2008).

[4] Yan, H. *et al.* Damping pathways of mid-infrared plasmons in graphene nanostructures. *Nature Photon.* **7**, 394-399 (2013).

[5] Mikhailov, S. A., Savostianova, N. A. Microwave response of a two-dimensional electron stripe. *Phys. Rev. B* **71**, 035320 (2005).

[6] Nikitin, A. Y., Low, T., Martin-Moreno, L. Anomalous reflection phase of graphene plasmons and its influence on resonators. *Phys. Rev. B* **90**, 041407 (2014).

[7] Burke, P. J., Spielman, I. B., Eisenstein, J. P., Pfeiffer, L. N., West, K. W. High frequency conductivity of the high-mobility two-dimensional electron gas. *Appl. Phys. Lett.* **76**, 745-747 (2000).

[8] Dyer, G. C. *et al*. Induced transparency by coupling of Tamm and defect states in tuable terahertz plasmonic crystals. *Nature Photon.* **7**, 925-930 (2013).
